# Intertemporal trade-off between population growth rate and carrying capacity during public good production

**DOI:** 10.1016/j.isci.2022.104117

**Published:** 2022-03-19

**Authors:** Manasi S. Gangan, Marcos M. Vasconcelos, Urbashi Mitra, Odilon Câmara, James Q. Boedicker

**Affiliations:** 1 Department of Physics and Astronomy, University of Southern California, Los Angeles, CA, USA; 2 Commonweath Cyber-Initiative and Bradley Department of Electrical Engineering, Virginia Polytechnic Institute and State University, Arlington, VA, USA; 3 Ming Hsieh Department of Electrical & Computer Engineering, Department of Computer Science, University of Southern California, Los Angeles, CA, USA; 4 USC Marshall School of Business, University of Southern California, Los Angeles, CA, USA; 5 Department of Biological Sciences, University of Southern California, Los Angeles, CA, USA

**Keywords:** Computational molecular modeling, Microbiology

## Abstract

Public goods are biomolecules that benefit cellular populations, such as by providing access to previously unutilized resources. Public good production is energetically costly. To reduce this cost, populations control public good biosynthesis, for example using density-dependent regulation accomplished by quorum sensing. Fitness costs and benefits of public good production must be balanced, similar to optimal investment decisions used in economics. We explore the regulation of a public good that increases the carrying capacity, through experimental measurements of growth in *Escherichia coli* and analysis using a modified logistic growth model. The timing of public good production showed a sharply peaked optimum in population fitness. The cell density associated with maximum public good benefits was determined by the trade-off between the cost of public good production, in terms of reduced growth rate, and benefits received from public goods, in the form of increased carrying capacity.

## Highlights

• Public good production creates trade-off between growth rate and carrying capacity
• Cell density-dependent regulation times the production to optimize this trade-off
• At this time, benefits of public good are maximum and received instantaneously

## Graphical abstract

**Figure:**
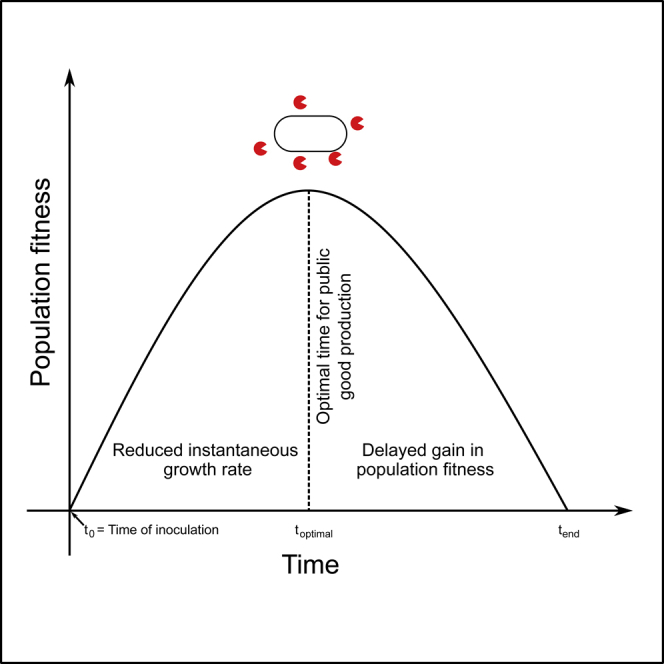

## Introduction

Often at higher cell densities, microorganisms favor cooperative interactions by producing metabolically expensive biomolecules known as public goods (Boyle et al., 2013; Nadell et al., 2008b; West et al., 2007). Important for growth and propagation, public good molecules are synthesized and secreted by cells into the extracellular environment and their activity is a benefit to both the producing cells and neighboring cells (Brockhurst et al., 2008; Drescher et al., 2014; Nadell et al., 2008a). In the context of proliferating bacterial communities, public good supports continued growth and population stability, and delaying its synthesis can eventually decelerate population growth (Özkaya et al., 2018). However, expression of public good imposes an immediate cost in terms of reduced intrinsic growth rate; in one example, growth was reduced by as much as 83% (Pai et al., 2012), thereby affecting the net benefit of the public good. Production and utilization of public goods in microbial communities, thus, give rise to an economy, whose interest lies not only in maximizing the rewards delivered by public goods but also reducing the energetic costs of public good production.

Economics recognizes this as a problem of optimization of trade-off between costs and benefits from production and consumption of goods. Theoretically, the populations that bear the consequences of public good production in real time are categorized in two groups. Populations that disregard future benefits from public good and prefer investing its resources to support growth in the present are identified as “impatient” populations. On the other hand, populations that desire future gratification rather than present satisfaction are called “patient” populations (Chapman, 1996). Both populations seem to suffer losses in terms of stagnant growth in the future or reduced intrinsic growth rate in the present, respectively. Under such situations, economics explains that an ideal time for investments in public good production can be determined by analyzing the payoffs obtained from public good production and then evaluating the cost imposed due to delayed investments (Frederick et al., 2002). This raises the question how biological systems account for the potential of a time-delayed benefit received from an energetically costly activity.

While production and extracellular secretion of public good molecules like bacteriocins, various chelating agents, and polymers employ specialized regulatory mechanisms to ensure the efficiency of the processes (Gillor et al., 2008; Rankin et al., 2011; Zhang and Rainey, 2013; Ross-Gillespie et al., 2015; Bayramoglu et al., 2017), bacteria control expression of some public good molecules through the process of quorum sensing (Brown and Johnstone, 2001; Nealson and Hastings, 1979). Quorum sensing involves the accumulation of an autoinducer signal, which in turn, enables cells to delay the production of public goods until reaching a high cell density (Drescher et al., 2014). Thus, populations of cells can use quorum sensing to coordinate the timing of public good production. Over the years, several studies have captured the important role of QS and non-QS regulatory mechanisms in maximizing the benefits collected from public good molecules. Several studies have captured the importance of quorum sensing to tune public good production with cell density (Bruger and Waters, 2016, 2018; Heilmann et al., 2015; Li et al., 2021; Pai et al., 2012; Schluter et al., 2016; Schuster et al., 2017; Brockhurst et al., 2008; Kümmerli and Brown, 2010). Prominent examples include QS-mediated tuning of public good production to increase population fitness in *Vibrio harveyi* (Bruger and Waters, 2016), or robustness of quorum sensing pathway itself at all initial densities ensuring population survival in long term (Pai et al., 2012). In 2010, Kummerli and Brown showed that facultative regulation of the energetically costly molecule pyoverdine based on iron availability in the growth medium *Pseudomonas aeruginosa* reduces the cost of production and decreases the ability of cheaters to overtake the population (Kümmerli and Brown, 2010). However, the ability of these regulatory processes to negotiate current costs, such as an immediate reduction in growth rate, with future gains, such as increased population fitness, remains to be analyzed. Selective pressure should favor a regulatory mechanism to continuously probe population density over time to optimize the timing of the production of public goods. Here, we pose quorum sensing as a process that helps an individual bacterial cell to express public good at an ideal time and to extract a maximum net benefit.

To test this idea, we designed a synthetic genetic circuit to quantify the time-dependent variation in cost and benefit received from the expression of a public good. The public good, an α-amylase enzyme, increases the supply of nutrients via digestion of starch. The expression of the public good was timed by the exogenous addition of a high concentration of inducer, in this case the quorum sensing signal 3-oxo-C6-acylhomoserinelactone. Our experiments show that time is a key aspect in the formulation of a population decision of committing to public good expression. Under given growth conditions, expression of public good at an optimal time point balances the trade-off between population growth at present and increased carrying capacity in the future. We corroborate our experimental results with theoretical studies by proposing a modified logistic growth model for populations that synthesize energetically expensive biomolecules. In our model, the intrinsic growth rate and carrying capacity change over time according to an activation signal regulated by quorum sensing. Our experimental data show that the switch between high and low growth rate and carrying capacity may not occur instantaneously. This potential latency in the change in the carrying capacity leads to an optimal population density to activate α-amylase expression.

## Results

### Population density-regulated expression of public goods optimizes the trade-off between population growth rate and carrying capacity

To experimentally test the fitness effects of public good production, we engineered a set of *Escherichia coli* strains to produce the public good α-amylase (Roy et al., 2013), an extracellular enzyme that digests polymeric starch into simpler sugars like glucose and maltose that can be absorbed and metabolized by the cells. The *amyE* gene encoding for α-amylase was copied from the genome of *Bacillus subtilis 168* and incorporated into a plasmid for heterologous expression in *E. coli* MG1655. Extracellular secretion of functional α-amylase in *E. coli* populations was confirmed, by growing cells on M9 agar containing starch as a sole source of energy. As detected by Lugol’s iodine test (Cochran et al., 2008), a distinct halo appeared around every growing colony implying extracellular expression of α-amylase and its subsequent utilization by bacterial population as a public good to digest starch into glucose molecules (Figure S3). On the plasmid, public good, α-amylase was regulated by a quorum sensing gene circuit. Cells express both the *luxI* and *luxR* genes, which synthesize and detect the autoinducer molecule 3-oxo-C6-acylhomoserinelactone (AHL). The entire circuit as well as the public good gene was regulated by the quorum sensing-responsive *P*_*lux*_ promoter. Using this circuit, the production of α-amylase is delayed until the concentration of AHL exceeded a threshold concentration. Cells were grown in M9 media containing both starch and a low concentration glucose, such that cells utilizing only glucose would grow initially and sustained growth would require public good production. Once activated, cells expressed α-amylase (Figure S2A), which increased the amount of growth within the population.

**Figure 1:**
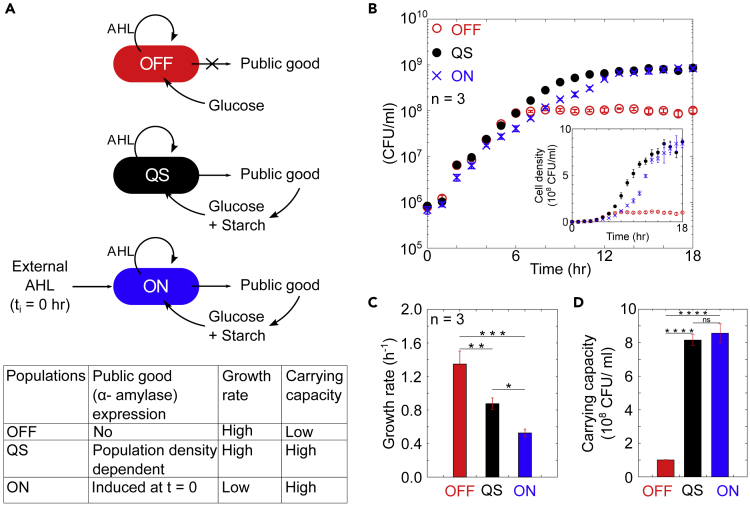
The cost and benefit of production of the public good α-amylase (A) Depiction of three different strategies for production of a public good, α-amylase. OFF cells cannot produce the public good, QS (quorum sensing) produces public good at high cell density, and ON cells are externally induced to produce public good at the beginning of the experiment (t_i_ = 0). Cells are grown in media with glucose and starch as carbon sources, such that α-amylase increases the amount of carbon available for cell growth. (B) Growth of cultures in the conditions of OFF (red open circles), QS (black filled circles), and ON (blue cross) populations. (C and D) Growth rates and (D) carrying capacities for the three regulatory strategies were calculated by fitting logistic growth equation to growth data. Observed differences in growth rates and carrying capacity across the three growth conditions were confirmed using one way ANOVA at 95% CI followed by Tukey HSD/ Tukey Kramer test (Tables S2 and S3). For all plots, n = 3 and error bars show SD.

To validate the basic cost and benefit associated with this production of the public good, we tested three growth strategies: OFF, ON, and quorum sensing (QS), as shown in Figure 1A. In the “OFF” strategy, *amyE* was deleted from the genetic circuit. “OFF” populations cannot hydrolyze starch and only metabolize glucose. “ON” cells contained the quorum sensing-regulated gene circuit and were stimulated through the addition of external AHL at the beginning of the experiment, such that the public good was produced continuously throughout the experiment. These cultures can consume free glucose and degrade starch as an additional food source. In the quorum sensing, “QS”, strategy, production of α-amylase was controlled via AHL autoinducer. As opposed to the “ON” condition, external AHL was not added at the beginning of the experiment, but instead AHL was produced by the cells and accumulated in the culture medium over time. Quorum sensing was activated at cell density around 10^7^ CFU/mL leading to public good production (Figures S4A and S4B)

The growth behavior of these three strains, OFF, ON, and QS, was measured to determine the advantages and disadvantages of each strategy. All three strains were inoculated into M9 media supplemented with glucose and starch. Presence of starch in the medium increases the turbidity, preventing accurate measurement of the cell density using light spectroscopy. Hence, we measured cell density over time by spreading aliquots of culture onto LB agar plates and counting colony-forming units. As shown in Figure 1B, growth dynamics differed between the three strains. The initial growth rates of “OFF” and “QS” were greater than the “ON” population, indicating an energetic cost associated with public good production. There was also a change in the carrying capacity of these three strains. Growth was slowed down for the OFF strain at approximately 10^8^ cells/mL. The QS and ON strains continued to grow until approximately 8 × 10^8^ cell/mL due to the ability of the public good to release additional nutrients from the media. Differences in the population growth patterns were quantified in terms of growth rate (Figure 1C) and carrying capacity (Figure 1D). Both the parameters were calculated by fitting logistic equation to the growth measurements. These metrics clarify the costs and benefits of public good production.

There is a clear cost to the public good, as demonstrated by reduced growth of the ON culture at early times. The benefit of amylase production under these culturing conditions is an increase in the carrying capacity, as shown in the ON and QS cultures. Our experiment demonstrated that for bacteria expending energy to gain an increased carrying capacity, the density-dependent regulation was a successful strategy to reduce the initial cost while maintaining the benefit of increased carrying capacity.

### The optimal delay to public good production maximizes population fitness

As shown in Figure 1, production of α-amylase has the benefit of increased carrying capacity at the cost of reduced growth rate. Regulating public good production via QS was more beneficial than both not producing the public good and always producing the public good. Next, we measured how the timing of public good production influenced population fitness.

**Figure 2:**
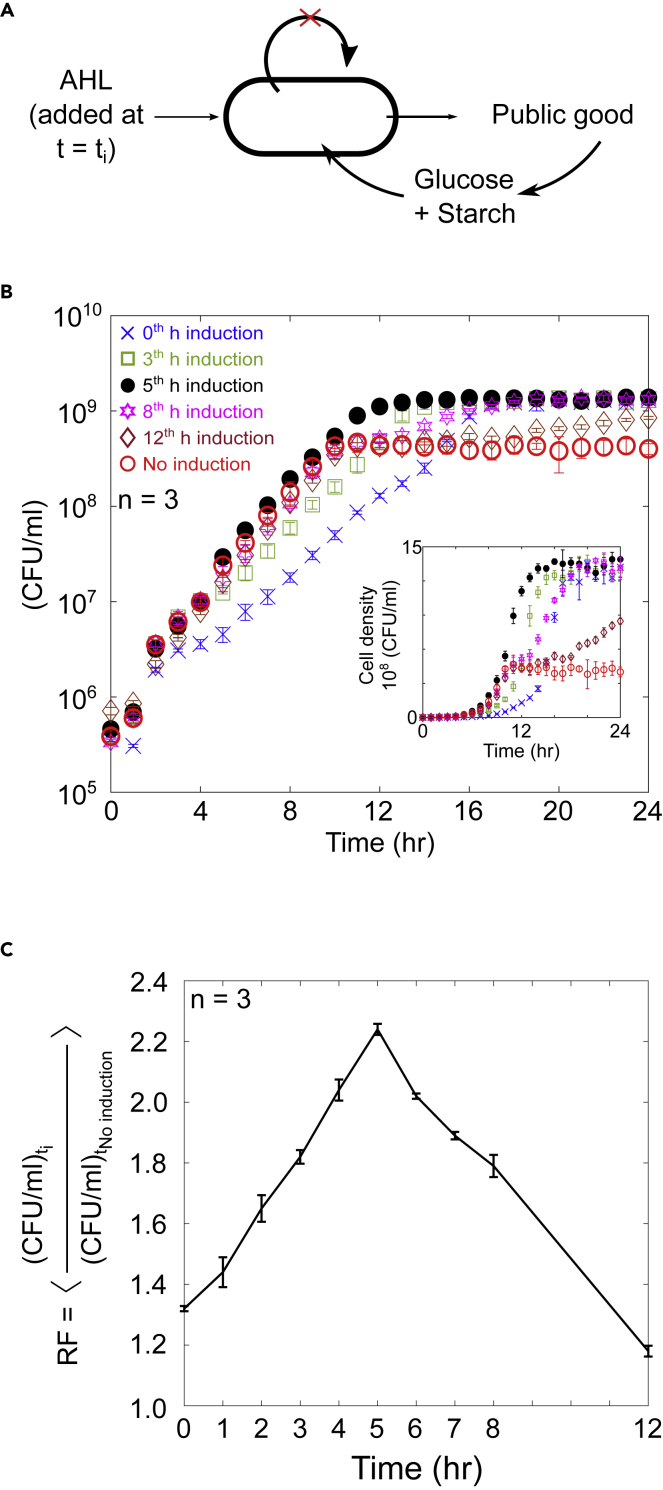
Finding the optimal time to initiate public good production (A) Schematic shows public good production when induced by external AHL at t = t_i_. Cells in these experiments do not synthesize AHL. (B) Growth of individual populations activated with AHL added externally at 0, 3, 5, 8, and 12 h. No induction shown as negative control. (C) Fitness of the induced cultures relative to the uninduced culture averaged over the entire experiment. The relative fitness is plotted as a function of the induction time. n = 3 for all measurements and error bars show SD.

To precisely regulate the timing of public good production, we created a new gene construct with a truncated *luxI* gene, thus cells could not produce the AHL inducer (Figures S1C and S5). Cells equipped with this circuit used glucose from the medium as carbon source until induction by externally added AHL. Induction led to expression of α-amylase and the extracellular digestion of starch into additional nutrients that could be used for growth (Figures 2A and S2C). In parallel cultures, production of the public good α-amylase was induced at different times (t_i_), and changes in cell density were monitored by plate counts.

As shown in Figure 2B, cultures that were induced at early times had slower initial growth, yet all induced cultures eventually reached the elevated carrying capacity. As the induction time was delayed, cultures maintained the higher growth rate for longer times. See Figure S6A for data from additional induction times. To find the optimal time to initiate production of α-amylase, the relative fitness (RF) of each culture was calculated (Figure 2C) by taking the ratio of cell density of a given culture to the uninduced negative control averaged over the entire experiment, from 0 to 24 h, as explained in (Equation 8) to reflect the changes in population growth rate as well as in carrying capacity. The maximum relative fitness was for induction at 5 h. Our results substantiate that an optimum time delay preceding the synthesis of a public good, maximized cell fitness at a specific point in a time, when trade-off between the growth rate and carrying capacity appears to be balanced. Deviation from this time point either imposes production cost on populations, causing reduction in the growth rate or limits nutrient resources resulting in diauxic growth. In both cases, total population fitness drops down below the optimum, due to which we observe a roughly bell-shaped curve when fitness is plotted as a function of production time. Next, a mathematical model explores how adjustment of the threshold parameter, the density at which activation occurred, influences cell growth and leads to an optimal time to initiate public good production.

### Developing a growth model that includes costs and benefits of public good production

**Figure 3:**
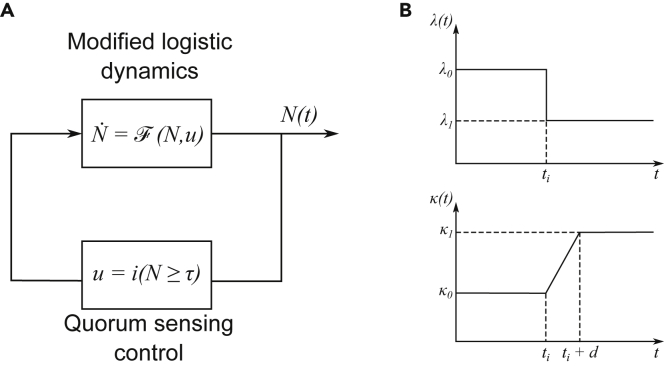
Developing a modified logistic growth equation to incorporate the cost and benefit of public good production (A) Block diagram of our system model for control of bacterial growth via quorum sensing. The state of the system is the number of cells, *N*. The control signal, *u*, corresponds to the decision of activating or not the production of a costly enzyme. The policy used to compute the control implements quorum sensing, where activation occurs once a certain target population is met. (B) Changes in the growth rate and carrying capacity over time. The decision to produce the public good is reached at t = i.

Our mathematical model is a modified version of the traditional logistic growth model:

(Equation 1) $$\dot{N}\left(t\right)=F\left(N\left(t\right),u\left(t\right)\right)$$

(Equation 2) $$F\left(N,u\right)=N\lambda \left(u\right)\left(1-\frac{N}{\kappa \left(u\right)}\right)$$

where N(t) denotes the cell density at time t. The logistic growth model is characterized by two parameters: the intrinsic growth rate (λ), and the carrying capacity (κ). As depicted in Figure 3A, the control signal that modifies the growth rate and carrying capacity of the colony is denoted by *u*. The control signal is a function of the concentration of the AHL autoinducer used to regulate expression of quorum sensing-responsive genes, here the public good α-amylase. For the measurements with the “QS” strain in Figure 1, this signal accumulates over time as a result of AHL synthesis by the cells. For the “ON” strain in Figure 1 and the externally induced strains used in Figure 2A, a high concentration of signal was added at time *t*_*i*_.

In the model, activation of quorum sensing, beyond time *t*_*i*_ or when the AHL concentration exceeds a threshold concentration for the “QS” strain, results in a switch in the value of both the carrying capacity and the specific growth rate. The growth rate is decreased due to the burden of public good production and the carrying capacity is increased as additional nutrients are now available for growth. We assume that the switch in the growth rate constant is instantaneous, as depicted in Figure 3B. The increase in the carrying capacity is not always instantaneous, as suggested by the results shown in Figure 3B when external signal was added at t = 12 h. Before 12 h, the culture had already reached the carrying capacity, and activation resulted in a slow drift of the population size from the lower carrying capacity toward the higher carrying capacity. The scheme for the gradual change in the carrying capacity is depicted in Figure 3B and incorporated into the model using:

(Equation 3) $$\begin{array}{lll} \kappa \left(t\right)=\{\kappa_{0} & t<t_{i}\;\kappa_{0}+\;\frac{\kappa_{1}-\kappa_{0}}{D\left(N\left(t_{i}\right)\right)}\left(t-t_{i}\right) & t_{i}\geq t\\ & \leq t_{i}+D\left(N\left(t_{i}\right)\right)\;\kappa_{1} & t>t_{i}+D\left(N\left(t_{i}\right)\right)\; \end{array}$$

*D* accounts for the time it takes to change from the smaller carrying capacity to the larger carrying capacity. This delay may have many contributions, including the time needed for enzyme production and sufficient starch degradation to impact cell growth.

If there is no delay $\left(D\left(N\left(t_{i}\right)\right)=0\right)$, then our model for a quorum sensing population simplifies to:

(Equation 4) $${\dot{N}}_{qs}\left(t\right)=\left(\lambda_{0}-\left(\lambda_{0}-\lambda_{1}\right)i\left(N_{qs}\left(t\right)\geq \alpha \right)\right)N_{qs}\left(t\right)\left(1-\frac{N_{qs}(t)}{\kappa_{0}-(\kappa_{0}-\kappa_{1})i(N_{qs}(t)\geq \alpha )}\right)$$

where the function i(S) denotes the indicator function of the argument S, i.e., i(S)=1 if *S* is true, and i(S)=0 if *S* is false.

### Fitting experimental data to calculate the delay in the benefit from public good production

Next, we implemented the model to calculate the values of the parameters in ((Equation 1), (Equation 2), (Equation 3)). Figure 2 reports a set of three independent time-series data for the population activated at times $t_{i}$ ranging from 0 to 12 h, including a culture that was not activated. Each series contains 25 samples, taken in 1-h long intervals. For simplicity, we have used a discrete-time approximation of the model in ((Equation 1), (Equation 2), (Equation 3)). To calibrate the model, the first step is to estimate the carrying capacity and the intrinsic growth rates for the ON and OFF cultures, t_i_ = 0 and no induction, respectively. This was done using a weighted nonlinear least-squares regression, in which we fixed the initial condition of our model N(0) as the average of the first sample in each of the three data series corresponding to the ON or OFF cultures . This step is necessary because the data vary by several orders of magnitude from the first sample to the last. Therefore, with a free initial condition as an optimization parameter, least-squares will ascribe a much larger weight to the steady-state regime, which leads to very good estimates of the carrying capacity, and poor estimates of the intrinsic growth rate. Similarly, a least-squares optimization in log scale creates the opposite problem resulting in an excellent estimate for the growth rate, but a poor estimate for the carrying capacity. Albeit atypical, the strategy of fixing the initial condition and performing regular least-squares fitting leads to good estimates for both quantities. Also notice that the growth rate and capacity were obtained for the discrete-time model, which was compared with the sample average of the data using least-squares as follows:

(Equation 5) $$min_{\kappa ,\lambda }\sum_{k=1}^{24}w\left(k\right)\;{\left(N\left(k\right)-N_{data,j}\left(k\right)\right)}^{2}$$

where the weights w(k) are the inverse of the SD of the measurements $N_{data,j}\left(k\right),\;j=1,2,3$.

**Figure 4:**
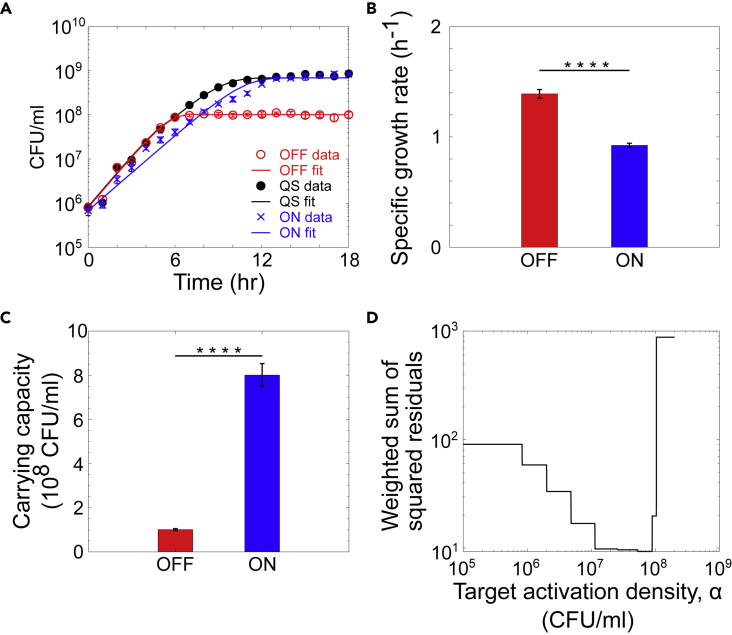
Fits to the modified logistic growth equation (A–C) Using the experimental data from the ON and OFF strategies reported in Figure 1B, best-fit values for the $\lambda_{0},\;\lambda_{1},\;\kappa_{0}\;$ and $\kappa_{1}$ were calculated. The difference between the parameters calculated for each condition was validated using unpaired t test at 95% CIs. The values for these parameters were used to predict the growth dynamics of the strain following the quorum sensing strategy described in Equation (4). For all plots, n = 3 and error bars represent SD. To find the target activation density $\alpha^{*}$, we solve another nonlinear weighted least-squares regression problem. For the quorum sensing strain, optimal activation threshold $\alpha^{*}$ = 5.3 10^7^ CFU/mL. (D) Weighted sum of the squared residuals as a function of α.

Best-fit values for κ and λ for the ON and OFF cultures are shown in Figure 4. The best-fit value of the specific growth rate for the OFF culture is much higher than the previously calculated value reported in Figure 1C, as Figure 1C shows the results of an exponential fit to the first six timepoints and Figure 4 shows the results from a fit to the logistic growth equation.

The second step in the data analysis was to estimate the delay in reaching the gain in carrying capacity for each of the induction times $t_{i}$. The procedure we used was slightly different than the one described above. For each activation time $t_{i}$, we perform a nonlinear least-squares optimization over the delay parameter *D*, from (Equation 3), and the initial condition N(0), as follows:

(Equation 6) $$\;min_{N\left(0\right),D}\;\overset{24}{\underset{k=1}{\sum }}w\left(k\right){\left(N\left(k\right)-N_{data}\left(k\right)\right)}^{2}$$

where N(t) is computed according to a discrete-time approximation of (Equation 1), (Equation 2), (Equation 3), and w(k) is a weight which is inversely proportional to the SD of the time-series data.

**Figure 5:**
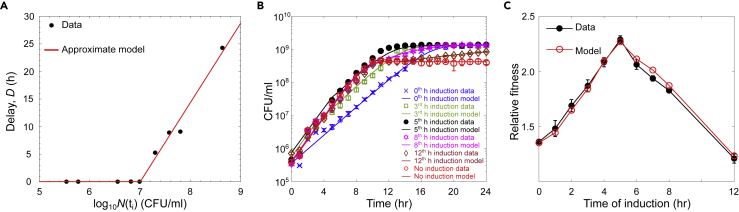
Fits for the delay in the benefit from public good production (A) From the experimental data, the best-fit value of the delay time was calculated for each induction time t_i_. (B) The red line is the piece-wise linear function describing the relationship between delay in carrying capacity as a function of the population at the activation time (B) The solid lines show the model prediction made after fitting for the delay parameter D. Symbols show experimental measurements. (C) Empirical and theoretical fitness function computed from data and our calibrated mathematical model, respectively, which shows that our simple model (with a minimal number of parameters) successfully captures the fundamental trade-off that occurs in bacterial growth controlled via QS. For all plots, n = 3 and error bars represent SD.

The reason why the joint optimization of N(0) and D works well in this case is that the growth rates and carrying capacities ($\lambda_{0},\;\lambda_{1},\;\kappa_{0},\;\kappa_{1}$) are now fixed, and the issue of the transient and stationary regimes dominating the objective function does not exist in this case. Using the best-fit values of the delay shown in Figures 5A and 5C compares the experimental data and to the model predictions.

As shown in Figure 5A, the highest population density at which there is no delay in receiving the public benefit from public good production is at approximately 1 × 10^7^ CFU/mL. There is a delay in the change in the carrying capacity if α-amylase production is initiated at cell densities above this range. As shown in Figure 5D, when using the best-fit parameters for the growth rates and carrying capacities and the delays in public good benefit for each activation density, we reproduce the relative fitness trend calculated from the experimental data. There is a density at which the initiation of public good production optimizes fitness, and this optimal density corresponds to a density for which there is no delay in the public good benefit. The penalty, or delay of the public good benefit, increased for cultures that initiated public good production further outside of this optimal range. These findings demonstrate that for α-amylase production as a public good, there is an optimal range of cell densities to initiate public good production. Nonlinear least-squares regression of the data for the quorum sensing strain in Figure 1 (“QS”) revealed activation of quorum sensing in the range of 5–9 × 10^7^ CFU/mL, which is very close to the optimal strategy for regulation of public good production.

The intrinsic growth rate and the carrying capacity obtained using the method described in the previous section are very close to the ones obtained using the nonlinear regression procedure herein. To obtain a consistent set of parameters, it is important to use the entire time-series data to estimate the growth rate. The joint optimization of growth rate and carrying capacity is desirable but not essential, since the carrying capacity can be accurately estimated using the last few samples of the growth curves.

## Discussion

In economics, decisions with consequences that play out in future are described by the concept of intertemporal choices (Berns et al., 2007). When making a decision, individuals tend to assign an additional cost to rewards received at a later time (Chapman, 1996; Frederick et al., 2002; Samuelson, 1937). This creates a specific point in time at which benefits of the decision exceed its cost, thereby maximizing the trade-off between the two. Personal goals of a decision maker play a crucial role in determining the ideal time for action (Samuelson and Zeckhauser, 1988).

The model considered herein captures the fundamental trade-offs observed in the experimental data. Importantly, it also accounts for the latency observed during activation past the typical values of activation observed in nature. One major distinction of our model with previous work in this area is that a reduction in growth rate leads to a gain in capacity. Previous works have not explored a switch in carrying capacity; see for example the work of Pai et al. (Pai et al., 2012).

For a bacterial cell, the decision to expend energy for public good production also has an intertemporal dimension. Cultures that activate too early bear the cost of public good production before these resources are needed, whereas waiting too long to produce public goods leads to diauxic growth, with a lag between glucose and starch utilization. Early production of public good at lower cell densities, shunts cellular energy into biochemical pathways rather than growth and reproduction. As a result, public good production compromises the instantaneous growth rate of the population. However, the strategy to stall public good production to support the current population growth rate, leaves populations unprepared for future growth conditions and eventually has a negative effect on population fitness (Heilmann et al., 2015; Levin, 2014; Pai et al., 2012; Özkaya et al., 2018). Postponing the expression reduces glucose content of the environment and reduces the growth until glucose is again made available. Here, we have studied quorum sensing as a process to set the intrinsic population growth rate while deciding an appropriate time for an activation of public good production, thereby optimizing the trade-off between population growth rate and carrying capacity continuously in time.

The optimal strategy measured here was to activate public good production at a cell density around 10^7^ CFU/mL, a typical density for upregulation of quorum sensing-responsive genes in many natural systems (Nealson et al., 1970). It is likely a coincidence that the optimal strategy for our synthetic system agreed with the typical quorum sensing behavior of real systems. The optimal timing of any system would be dependent on many factors that influence the density at which quorum sensing-regulated genes are overexpressed and the cost and benefit of the public good. For example, factors such as the gene copy number and properties of quorum sensing-regulated promoters should modulate the dynamics of public good production (Wellington and Greenberg, 2019). The cost and benefit of public good production would depend on expression dynamics and the conditions in which the public good is acting, for example how much starch is present. Factors such as transport dynamics of the signal, characteristics of both the signal and receptor, and expression level of the synthase protein would also modulate how public good production depended on cell density. Together, these factors, and likely others, would set an optimal strategy for each system. Given that in these experiments cells used a wild-type quorum sensing system and a public good with characteristics similar to many extracellular enzymes, it may not be too surprising that the optimal timing for our synthetic system closely matched the expected density for upregulation of quorum sensing-regulated genes.

Earlier studies that discuss the trade-off between population growth rate and carrying capacity, emphasize on the tendency of the populations to use catabolic pathways associated either with fast but lower ATP output, inefficient catabolism, or with slower but higher ATP yield, efficient catabolism (Bachmann et al., 2013; Luckinbill, 1978; Pfeiffer et al., 2001). Because population growth is dependent on ATP production, use of either of the metabolic pathways impacts the trade-off. Inefficient ATP production has been shown to increase population growth rate by increasing division rate of a single cell and reducing the biomass yield. Efficient ATP production is known to hamper the growth rate of single cell while eventually increases the average population fitness (Pfeiffer et al., 2001). This phenomenon is also known as tragedy of commons (MacLean, 2008), that acts as a deciding factor in trade-off between population growth rate and carrying capacity. Further efforts in the same field have confirmed the role of this trade-off in shaping the evolution in bacteria. Prominent example could be the study conducted by Maclean and Gudelj in 2006, in which, coexistence of two different yeast strains, indulging either in efficient or in rapid ATP production. The observation was attributed to the density-dependent accumulation of toxic byproducts of a rapid ATP-producing biochemical pathway that inhibits the growth of the strain using the same pathway and favors other strain that produces ATP efficiently (MacLean and Gudelj, 2006). The work hints at the importance of population density in strategizing resource utilization. With this work, we are attempting to explore the cell density-dependent weighing of costs and benefits of public good production through time that culminates in optimizing the trade-off between instantaneous population growth rate and carrying capacity for public good synthesizing populations.

Here, modeling of the transition to the utilization of starch as a nutrient has quantified the delay in a population reaping the benefit of the public good α-amylase. The model was adapted from previous work on quorum sensing as an optimal control system. Previous work has shown that under the assumption of perfect observation of the state of the system, namely, the population at a given time, a threshold policy on the population maximizes a discounted objective function. Later, this discrete time model (Vasconcelos et al., 2018) was extended to continuous time (Vasconcelos et al., 2019). More importantly, while assuming a more realistic model with partial observations of the state of the colony via realistic AHL signal dynamics, the discounted objective function was numerically shown to be unimodal in the target population threshold. Similarly, precise timing of public good production was shown here to optimize fitness. If a population activates α-amylase expression at too low of a cell density, there is a delay in the time between α-amylase production and the time at which the population requires starch degradation to maintain growth. There is also a delay in the benefit from the public good if production of α-amylase occurs after growth has begun to slow. This delay represents a lag in the ability of cells to metabolize starch degradation products, and the lag increases as public good production is further delayed. These two delays in realizing the benefit from α-amylase production creates an optimal cell density of public good production.

Prior work has established the important role of mass transfer in the benefit of shared public goods and quorum sensing regulation (Redfield, 2002; West et al., 2012; Hense et al., 2007; Cornforth et al., 2014). Though our study did not directly address molecular transport, the rates of mass transfer likely influence the optimal time for public good production to maximize the trade-off between instantaneous population growth rate and carrying capacity. Diffusivity of shared public goods should modulate the benefit received from starch degradation, which contributes to setting the optimal density at which bacterial populations activate public good production.

Over the years, several studies have considered the importance of quorum sensing as a control mechanism for public good production (Darch et al., 2012; Heilmann et al., 2015; Pai et al., 2012; Schluter et al., 2016; Schuster et al., 2017; Zhao et al., 2019; Özkaya et al., 2018; Eldar, 2011). Work done by You and colleagues has used a similar framework to establish the optimal regulation of quorum sensing. This study follows the growth of synthetic strain of *E. coli* that expresses public good to survive under antibiotic stress. Though this work does not discuss the changes in the carrying capacity with public good production, it shows that the optimal population growth rate is possible only when public good production is switched on through quorum sensing (Pai et al., 2012). In our system, the cost of production of public good is reflected in the growth rate and the public benefit is the net gain in carrying capacity. Growth models where the individuals control the colony’s carrying capacity were previously studied in the social sciences in the work of Meyer and Ausubel (Meyer and Ausubel, 1999), where the adoption of a new strategy may create newly available resources that allow for higher population yield. Analogously to the bacterial case at hand, the adoption of a new strategy is a gradual process and does not yield immediate gains in carrying capacity. In other words, there is an inherent delay, especially if the population is already large. The work of de Vos et al. (de Vos et al., 2017) considered how interactions between different strains in a polymicrobial infection affect the growth rate and the carrying capacity. However, this change in behavior was passive rather than active, as in our model.

In conclusion, here, a bacterial population was shown to optimize the timing of public good production, such that there is no delay in receiving the benefit from the public good amylase. This optimal timing accounts weighs the short-term changes in the intrinsic growth rate with the long-term goal of maximizing the carrying capacity. Comparison of the regulatory strategies for public good production in different bacterial species may reveal how individual species have navigated such trade-offs in short-term and long-term costs and benefits. The cost and benefit of specific public goods should vary and will depend on environmental conditions, so perhaps such regulatory strategies will have evolved to be beneficial for typical environmental contexts. Certainly, evolutionary forces have the ability to adjust parameters such as the threshold signal concentration, or likewise the cell density of achieving a quorum. Incorporating the potential for reduction for future benefits due to time delays into such analysis may reveal new aspects of how populations of cells anticipate future conditions and evolve optimal regulatory strategies.

### Limitations of the study

Although the experiment herein is within the context of the quorum sensing (QS)-controlled production of public goods that directly affect growth by changing the cell’s metabolic behavior, similar trade-offs may exist in other contexts, which our model does not cover. Instead, our model addresses the case of a population whose QS mechanism can be used to explicitly regulate the trade-off growth rate vs. carrying capacity to maximize fitness. There are many other QS-regulated phenomena such as biofilm formation, virulence attacks, and motility. However, some of those phenomena do not result in a significant change in carrying capacity. Therefore, our trade-off does not apply to those cases. Nevertheless, it would be interesting to investigate which other QS-regulated phenomena result in significant gains in carrying capacity and how well our model would apply to those cases.

## STAR★Methods

### Key resources table

**Table:** 

REAGENT or RESOURCE	SOURCE	IDENTIFIER
**Bacterial and virus strains**		

*E. coli* MG1655	Tran and Boedicker, 2019	N/A
*E. coli* DH5α	NEW ENGLAND BioLabs®_Inc_	C2987H
*B. subtilis* 168	ATCC	23857™

**Chemicals, peptides, and recombinant proteins**		

Luria- Bertani Broth	Difco™	244610
M9 minimal salts, 5X	Difco™	248510
D- Glucose (Dextrose) Anhydrous	aMResco®	0188-1KG
Starch, soluble	SIGMA- ALDRICH	S9765-250G
N-(3-Oxohexanoyl)-L-homoserine lactone	Chemodex	143537-62-6
Iodine	SIGMA- ALDRICH	207772-5G
Potassium iodide	SIGMA- ALDRICH	207969-100G
Kanamycin sulfate	aMResco®	0408-25G
Phosphate Buffer Saline	VWR	E404-200TABS

**Deposited data**		

Simulation data	This paper	https://github.com/mullervasconcelos/QS-iScience-22

**Oligonucleotides**		

Primer: amyE_Fwd: 5′-TCACCTCGA GTCAATGGGGAAGAGAACC-3′	This paper	N/A
Primer: amyE_Rev: 5′-AGAGATGGG TATGTTTGCAAAACGATTCAAAAC-3′	This paper	N/A
Primer: pTD103_Fwd: 5′-TTGCAAACATA CCCATCTCTTTATCCTTAC-3′	This paper	N/A
Primer: pTD103_Rev: 5′-TCCCCATTGAC TCGAGGTGAAGACGAAAG-3′	This paper	N/A
Primer: ΔamyE_Fwd: 5′-ACCCAT CTCTTTATCCTTAC-3′	This paper	N/A
Primer: ΔamyE_Rev: 5′-ACCC ATCTCTTTATCCTTAC-3′	This paper	N/A
Primer: ΔluxI_Fwd: 5′-CTCAAAGATA AATACTCTGCTAGTGAAATTACAA-3′	This paper	N/A
Primer: ΔluxI_Rev: 5′-GACTTAGAATAC CTTTATACTCCTCCGATGG-3′	This paper	N/A

**Recombinant DNA**		

pTD103*luxI*_*sfGFP*	addgene	#48885
pPG_*amyE*^*+*^	This study	N/A
pPG_*amyE*^*-*^	This study	N/A
pPG_*amyE*^*+*^_*ΔluxI*	This study	N/A
pTD103*luxR*_*RFP*	Silva et al., 2017	N/A

**Software and algorithms**		

MATLAB	MathWorks®	R2020a
GraphPad Prism 9	GraphPad software	N/A
Inkscape-0.92.5-x64	INKSCAPE	N/A
ApE A plasmid editor	ApE A plasmid Editor by M. Wayne Davis	N/A

**Other**		

C1000 Touch™ thermal cycler	*BIO-RAD*	1851148
Infinite® M200PRO	TECAN	N/A

### Resource availability

#### Lead contact

Further information requests should be directed to the lead contact, Dr. James Q. Boedicker (boedicke@usc.edu).

#### Materials availability

This study did not generate new reagents.

### Experimental model and subject details

#### Strains and culturing conditions

*E. coli* MG1655 cells (Tran and Boedicker, 2019) were used as a parent strain for the propagation and expression of variations of the genetic circuit. Cultures were grown at 37°C at 200 rpm in M9 media supplemented with a carbon source and 50 μg/mL Kanamycin for plasmid maintenance. Primary cultures were grown overnight in M9 (Difco) media supplemented with 4% glucose (aMResco).

#### Plasmid construction

Genetic circuits for this study were designed by modifying pTD103*luxI*_*sfGFP* plasmid (Prindle et al., 2012) to test three strategies of α-amylase production that included ON (constitutive expression), QS (density-dependent expression) and OFF (no expression). Gibson assembly was used for plasmid construction (NEB). *amyE* gene encoding for α-amylase was amplified from genomic DNA of *B. subtilis 168* (ATCC 23857) and cloned into pTD103*luxI*_*sfGFP* under the regulation of the *P*_*lux*_ promoter, creating plasmid pPG_*amyE*^*+*^ used for measurements of the ON/QS strategy. The plasmid used for the OFF strategy, pPG_*amyE*^*-*^ was created by deleting the *amyE* ORF from plasmid pPG_*amyE*^*+*^. See Figures S1A and S1B.

For experiments to test the timing of public good production on growth dynamics, *luxI* gene was partially deleted from plasmid pPG_*amyE*^*+*^ to create plasmid pPG_*amyE*^*+*^_*ΔluxI*, to deactivate acetyl transferase activity of LuxI protein (Zaghlool and Al-Khayyat, 2015). Strains harboring this plasmid can respond to but do not synthesize the QS signal, therefore activation of public good production was induced by adding an external stimulus of signal. See Figures S1C and S5.

### Method details

#### Growth conditions

##### Optimization of trade-off between population growth rate and carrying capacity

To test the growth of cells using the ON, OFF, and QS strategies, primary cultures were diluted 1000X in 5 mL fresh media. For ON cells, public good production was induced by adding 3 μg/mL of 3-oxo-C6-acylhomoserinelactone (AHL, Adipogen) as in (Wellington and Greenberg, 2019). Upon reaching OD_600nm_ of 0.3, cells were washed three times 1X PBS (VWR, life science) and resuspended in 1 mL 1X PBS. Cells at 100X dilution were then inoculated into 5 mL fresh M9 media supplemented with 0.0125% glucose and 0.05% starch (Sigma- Aldrich). For the ON cells, 3 μg/mL of AHL was used to induce public good production. The cell density within triplicate cultures was monitored every hour for 18 h by counting colony forming units on LB agar with 50 μg/mL kanamycin.

##### Time dependent activation of public good

Primary and secondary cultures of cells harboring plasmid pPG_*amyE*^*+*^_*ΔluxI* were grown using the procedure for the QS strategy. Cells from the secondary culture were used to inoculate M9 media supplemented with 0.0125% glucose and 0.05% starch at 100X dilution. Using this time point as t = 0, 3 μg/mL of 3-oxo-C6-acylhomoserinelactone was added at the induction time. For cells labeled “no induction”, no external signal was added. The cell density within triplicate cultures was monitored every hour for 24 h by counting colony forming units on LB agar with 50 μg/mL kanamycin. For these experiments, cells induced at t = 0 were prepared following the procedure for the ON strategy described above.

#### Confirmation of α-amylase production and activity

100 μL of primary culture of *E. coli* MG1655 propagating pPG_*amyE*^*+*^ plasmid was spread on M9 minimal agar plate containing 0.5% starch (Sigma-Aldrich) as a carbon source and 50 μg/mL Kanamycin (aMResco®) as a selecting agent. The plate was incubated at 37°C for 3 days until visible colonies were formed. The entire plate was then flooded with Lugol’s reagent (Cochran et al., 2008) to observe halo around each colony confirming starch digestion by α-amylase secreted by bacterial cells, see Figure S3A.

1 mL of supernatants isolated from overnight grown cultures of (i) *E. coli* MG1655, (ii) *E. coli* MG1655 + pPG_*amyE*^*+*^ and (iii) *E. coli* MG1655 pPG_*amyE*^*-*^ were mixed with 0.05% Starch solution for 5 hours at 37°C, in an individual experiment. After 5 hours all the three solutions were tested for starch degradation using Lugol’s iodine reagent (Cochran et al., 2008), see Figure S3B.

#### Quantification of autonomous temporal production of 3-oxo-C6-acyl homoserine lactone by pPG_*amyE*^*+*^ cells

Cultures of *E. coli* MG1655 with pPG_*amyE*^+^ were prepared in 100 mL M9 supplemented with 0.0125% glucose and 0.05% starch using growth conditions identical to QS strategy. Growth was monitored for 18 hrs in terms of CFU/mL. At the end of each hour 1 mL culture was withdrawn and used for harvesting supernatant. Supernatants isolated for 19 timepoints were then added individually to *E. coli* cultures with pTD103*luxR*_*RFP* plasmids, also called as receiver cultures (Silva et al., 2017)*.* These cultures incubated for 4 hrs to OD_600nm_–0.5. Receiver cells were processed and quantified for RFP fluorescence using a microplate reader (TECAN, infinite M200PRO). Fluorescence values were compared with the standard curve obtained by treating parallel cultures of *E. coli* with pTD103*luxR*_RFP with different concentrations of commercially purified 3-oxo-C6-acyl homoserine lactone ranging from 10^−2^ to 10^4^ nM to extrapolate the concentration of 3-oxo-C6-acyl homoserine lactone produced by *E. coli MG1655* pPG_*amyE*^*+*^ cells over 18 hrs. See Figure S4A.

### Quantification of LuxI activity

*E. coli MG1655* strains bearing either pPG_*amyE*^*+*^or mutated pPG_*amyE*^*+*^_*ΔluxI* circuit were grown overnight in LB (Difco™) at 37°C. Supernatant collected from these cultures was then individually added to 200 μL liquid suspensions of *E. coli* cells containing pTD103*luxR*_*RFP* (Silva et al., 2017). Changes in RFP fluorescence was then monitored over 15 hrs using a microplate reader, see Figure S5.

### Quantification and statistical analysis

Population growth rate and carrying capacity were measured by fitting logistic equation (Equation 7) to the growth curves and extracting values for ‘r’ (growth rate) and ‘k’ (carrying capacity).

(Equation 7) $$y=\frac{Nk}{\left(N+\left(k-N\right)e^{-rt}\right)}$$

To calculate relative fitness of an induced cultures, the mean cell density of the induced culture over 24 hrs was divided by the mean cell density of the uninduced cultures, using:

(Equation 8) $$\;RF=\overset{24}{\underset{0}{\int }}\frac{N\left(t\right)}{N_{off}\left(t\right)}dt\approx \overset{24}{\underset{k=0}{\sum }}\frac{N\left(k\right)}{N_{off}\left(k\right)}$$

This definition is appropriate because despite the fact that the transient and stationary regimes are orders of magnitude apart, they are cancelled in the relative ratio inside the integral. Therefore, the integrand has approximately the same order of magnitude across time.

Difference between growth rate and carrying capacity calculated for experimental growth data observed in OFF, QS and ON conditions was confirmed with one- way ANOVA at 95% confidence interval followed by Tukey HSD test (refer to Tables S2 and S3), while unpaired t- test at the confidence intervals of 95% was used to show difference in growth rates and carrying capacities obtained for the fits after applying logistic regression to ON and OFF conditions as shown in Figure 4.

## Data Availability

• All the data has been included in file Data S1. • All original code has been deposited at GitHub and is publicly available as of the date of publication. DOIs are listed in the key resources table. • Any additional information required to reanalyze the data reported in this paper is available from the lead contact upon request.

## Supplemental information

Document S1. Figures S1–S6 and Tables S1–S3

Data S1. Raw data for population growth over time
