# Supplementary material for: Intertemporal trade-off between population growth rate and carrying capacity during public good production
Source: iScience. 2022 Mar 19;25(4):104117. doi: 10.1016/j.isci.2022.104117 (PMC8980746; doi:10.1016/j.isci.2022.104117)
Supplement: Document S1. Figures S1–S6 and Tables S1–S3 [file mmc1.pdf]

iScience, Volume *25*

Supplemental information

Intertemporal trade-off between population growth rate and carrying capacity during public good production

Manasi S. Gangan, Marcos M. Vasconcelos, Urbashi Mitra, Odilon Câmara, and James Q. Boedicker

Supplemental information

Supplemental tables and figures:

No.	Plasmid name	Use	Reference
1	pTD103*luxI_sfGFP*	Original plasmid	(Prindle et al., 2012)
2	pPG_*amyE*$^+$	Testing ON and QS strategy	This study
3	pPG_*amyE*$^-$	Testing OFF strategy	This study
4	pPG_*amyE*$^+$_*ΔluxI*	Time dependent induction	This study
5	pTD103*luxR_RFP*	AHL signal quantification	(Silva et al., 2017)

Table S1: Plasmids used in this study. Related to Figure 1, 2 and STAR methods.

Pair	Difference	SE	Q	Lower CI	Upper CI	Critical Mean	P- value
OFF- QS	0.486667	0.074266	6.539532	0.163411	0.807923	0.322256	0.008575
OFF- ON	0.844900	0.074266	11.376623	0.522644	1.167156	0.322256	0.000484
QS- ON	0.359233	0.074266	4.837091	0.036977	0.681489	0.322256	0.032642

Table S2: Statistical analysis of population growth rate calculated for ON, QS and OFF populations. One way ANOVA (95% confidence interval) followed by Tukey HSD test for growth rates calculated for experimental data obtained for ON, QS and OFF conditions. Related to Figure 1.

Pair	Difference	SE	Q	Lower CI	Upper CI	Critical Mean	P- value
OFF- QS	7.4 * 108	2.7 * 107	26.95395	6.2 * 108	8.5 * 108	1.2 * 108	3.0 * 10-6
OFF- ON	7.8 * 108	2.7 * 107	28.47771	6.6 * 108	9.0 * 108	1.2 * 10	2.2 * 10-6
QS- ON	4.2 * 108	2.7 * 107	1.523760	-7.7 * 108	1.6 * 108	1.2 * 10	0.560593

Table S3: Statistical analysis of carrying capacity calculated for ON, QS and OFF populations. One way ANOVA (95% confidence interval) followed by Tukey HSD test for carrying capacities calculated for experimental data obtained for ON, QS and OFF conditions. Related to Figure 1.

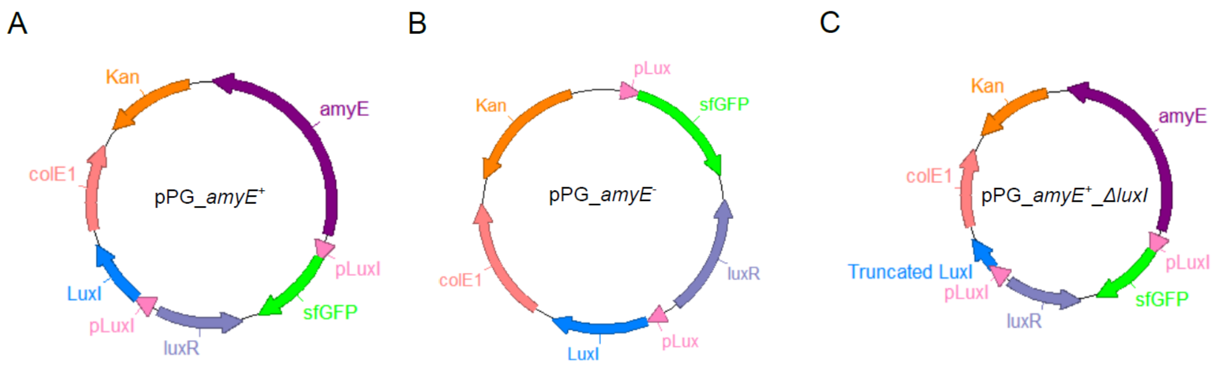

Figure S1: Schematic representation of plasmids used in this study. Plasmid maps of (A) pPG_*amyE*+ (B) pPG_*amyE*- and (C) pPG_*amyE*+_*ΔluxI*. Related to Figure 1 and 2 and STAR methods.

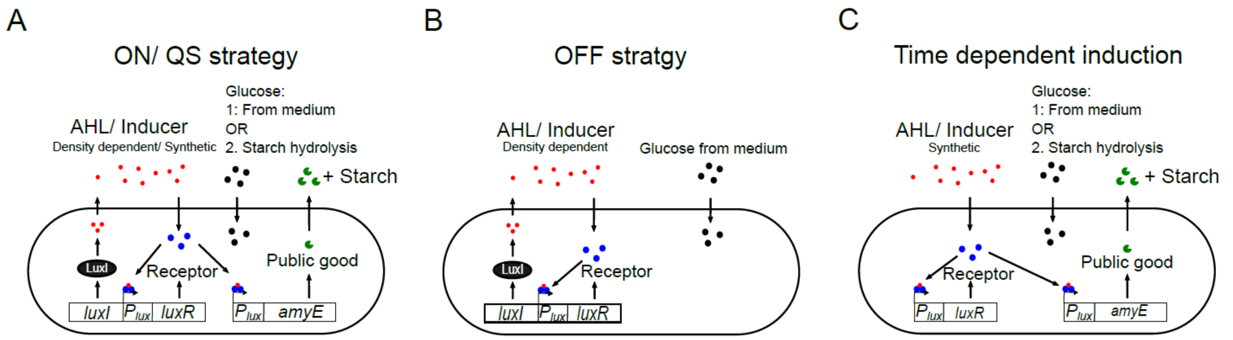

Figure S2: Schematic representations of different public good production strategies. Cartoons show (A) ON/ QS strategy (B) OFF strategy and (C) Time dependence to activate production of public good have been shown. Related to Figure 1 and 2.

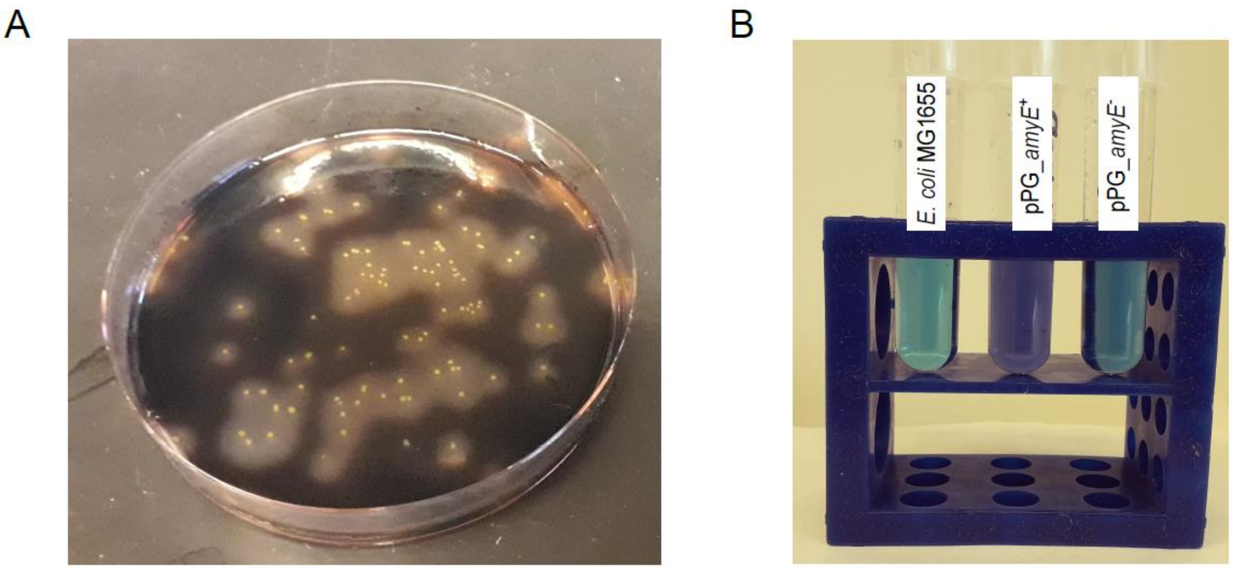

Figure S3: Confirmation of α- amylase production by *E. coli* cells. (A) Expression of α-amylase by *E. coli* cells propagating pPG_*amyE*$^{+}$ was confirmed by growing cells on M9 containing starch as a sole carbon source. Extracellular expression of α-amylase by cells digests starch into glucose/ maltose, thereby creating halo around colony upon iodine treatment indicating absence of starch. (B) Liquid cultures of *E. coli* cells transformed either with pPG_*amyE*$^{+}$ (middle tube) or with pPG_*amyE*$^{-}$ (right tube) were added to M9 supplemented with starch. Left tube shows host strain control. After incubation at 37°C and 200 rpm shaking for 2 days, medium was tested for starch content using Lugol's iodine and compared to an empty host. Related to STAR methods.

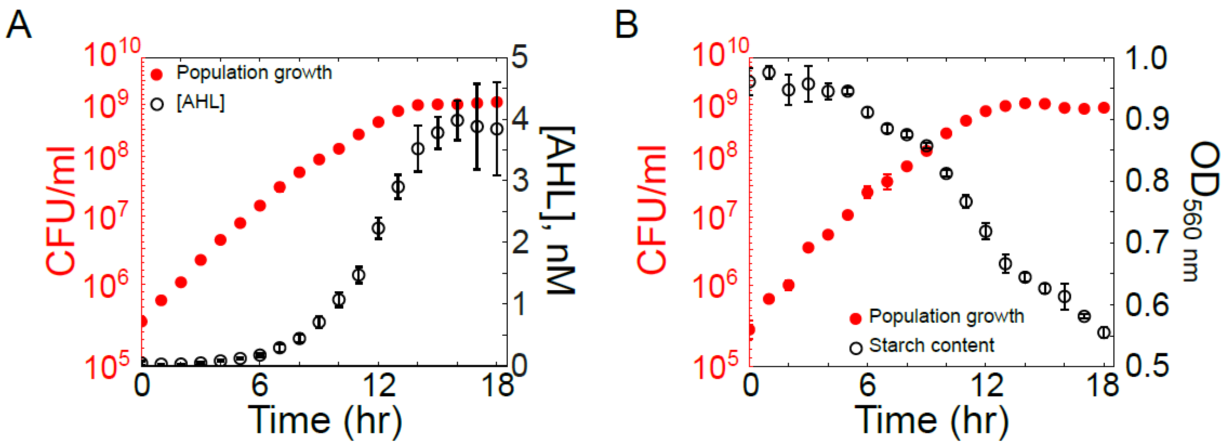

Figure S4: Signal production and amylase activity over time. pPG_*amyE*$^{+}$ cultures were grown under growth conditions identical to QS strategy for 18 hrs (red filled circles). Supernatant isolated from these cultures at every hour was quantified for (A) autonomous production of 3-oxo-C6-acyl homoserine lactone (AHL) and (B) starch content of medium (black open circles). Experiment was run in triplicates and error bars indicate the standard deviation across the samples. Related to Figure 1.

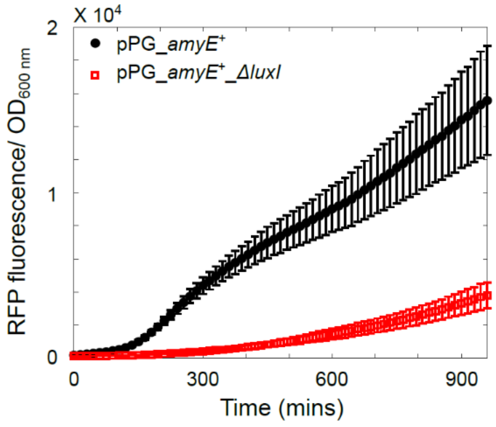

Figure S5: *luxI* mutant does not induce expression of quorum sensing regulated genes. To confirm the dysfunctionality of pPG_*amyE*$^+$_*ΔluxI* circuit, supernatant from overnight grown culture was added to the receiver circuit expressing RFP under P_{lux} promoter (Silva et al., 2017). Change in RFP fluorescence (red open squares) was monitored over time and compared to a culture that received supernatant from a signal producing strain harboring plasmid pPG_*amyE*$^+$ (black filled circles). n = 3. Error bars- standard deviation. Related to Figure 2.

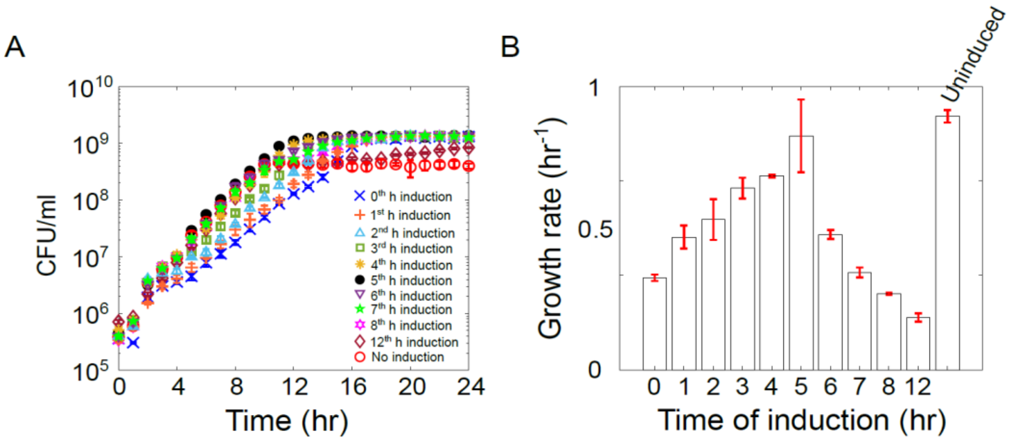

Figure S6: Extended data set for public good production induced between 0 and 12 hours. (A) Data for t=0, 3, 5, 8, and 12 h and the no induction control is identical to data from Figure 2b. (B) Respective growth rate of an individual population activated at different time for public good production has been calculated by fitting logistic equation to growth curves. n = 3. Error bars show standard deviation. Related to Figure 2.
